# Immunohistochemical Characterization of PepT1 and Ghrelin in Gastrointestinal Tract of Zebrafish: Effects of *Spirulina* Vegetarian Diet on the Neuroendocrine System Cells After Alimentary Stress

**DOI:** 10.3389/fphys.2018.00614

**Published:** 2018-05-24

**Authors:** Patrizia Lo Cascio, Concetta Calabrò, Clara Bertuccio, Carmelo Iaria, Fabio Marino, Maria G. Denaro

**Affiliations:** Department of Chemical, Biological, Pharmacological and Environmental Sciences, University of Messina, Messina, Italy

**Keywords:** immunohistochemistry, PepT1, GHR, zebrafish, digestive tract, Spirulina sp.

## Abstract

Gastrointestinal function in vertebrates is influenced by stressors, such as fasting and refeeding, different types of diet and hormonal factors. The aim of this paper was to analyze the effect of a Spirulina (*Arthrospira platensis*) diet, a microalga known for its nutraceutical properties, on the gastrointestinal tract of zebrafish (*Danio rerio*) regarding expression of oligopeptide transporter 1 (PepT1) and ghrelin (GHR). Food deprivation and refeeding was investigated to elucidate expression of PepT1 and GHR at a gastrointestinal level and the zebrafish compensatory mechanism. PepT1 is responsible for absorbing di- and tripeptides through a brush border membrane of intestinal mucosa. GHR is a brain-gut peptide in fish and mammals, stimulating growth hormone secretion and regulating appetite. Samples were taken after 2 and 5 days of specimen fasting, and 2 and 5 days of refeeding with Sera Spirulina tabs, in which the major constituent is Spirulina sp. (50.2% protein). Morphological and immunohistochemical analysis of PepT1 and GHR were carried out. Control specimen intestinal tract showed normal morphology of the digestive tract. Fasting caused fold structural changes and intestinal lumen constriction. Immunohistochemical analysis showed a PepT1 level reduction after fasting and an increase after refeeding, reaching very high levels after 5 days, compared to controls. GHR levels increased after food deprivation and gradually decreased after refeeding. Increased expression of PepT1 in refeeding fish suggests a compensatory physiological mechanism, as does the increase in GHR levels in fasting fish followed by a reduction after refeeding. A compensatory mechanism may be induced by fasting and refeeding and by a higher protein Spirulina diet. The microalga, for its nutraceutical properties, is an excellent candidate for animal breeding and human diet.

## Introduction

It is well known that teleosts in captive conditions are subject to stress phenomena, which may be due to illness or other factors, such as handling, transport, thermal variations, inadequate nutrition, exposure to periods of fasting or different types of diet and harmful agents. Several studies have documented how certain experimental procedures can influence gastrointestinal morphological integrity (Krogdahl and Bakke-McKellep, 2005; Denaro et al., 2006; Lo Cascio et al., 2017), metabolism, change of the enzymatic patterns (Caruso et al., 2011, 2012, 2014; Abolfathi et al., 2012), regulation of gastrointestinal hormones, and peptides transport (Jonsson et al., 2006; Olsson et al., 2008). Studies in zebrafish (*Danio rerio*, Hamilton, 1822), based on short term food deprivation and re-feeding to evaluate mRNA expression of the oligopeptide transporter (PepT1) and cholecystokinin (CCK), gastrin-releasing peptide (GRP) and ghrelin (GHR), showed that with successive refeeding PepT1 mRNA expression increased reaching values 8 times higher than pre-fasting levels (Koven and Schulte, 2012). PepT1 is a hydrogen ion/peptide cotransporter; it transports oligopeptides but cannot transport peptides with more than three amino acid residues or free amino acids. Its transport action requires proton binding and a negative membrane potential. PepT1 is a 708-amino acid protein with 12 putative membrane-spanning domains. It can absorb a part of ingested protein into the mucosa as di- and tripeptides. GHR is a 28-amino acid peptide, first identified and isolated from the stomach of rats (Kojima et al., 1999), which functions as a neuropeptide (Dickson et al., 2011). It is produced in the gastrointestinal tract by ghrelinergic cells (Sakata and Sakai, 2010) when the stomach is empty, conversely, secretion stops at full stomach. Besides regulating appetite, GHR also plays an important role in regulating energy distribution and rate (Burger and Berner, 2014). Its action on hypothalamic brain cells increases hunger, gastric acid secretion, and gastrointestinal motility (Nakazato et al., 2001; Shousha et al., 2005). It is highly conserved among species (Kaiya et al., 2011). Immunoreactivity of GHR was recognized in the GI tract and accessory organs of several species (Hayashida et al., 2002; Zhao and Sakai, 2008; Kasacka et al., 2014). GHR producing cells were found in the proventriculus, gizzard, and intestine of avians (Wada et al., 2003; Shao et al., 2010) and in stomach and intestinal epithelia of teleosts (Feng et al., 2013), as well as in molluscs (Ngernsoungnern and Ngernsoungnern, 2016). The GHR gene has been characterized in *Carassius auratus* (Unniappan et al., 2002), *Oreochromis mossambicus* (Kaiya et al., 2003), *Danio rerio* (Amole and Unniappan, 2009), and *Thunnus orientalis* (Suda et al., 2012). GHR mRNA is mainly expressed in fish stomach and intestine, but it has also been detected in the hypothalamus, spleen, and gill (Unniappan et al., 2002; Amole and Unniappan, 2009). Zebrafish (*Danio rerio*) is a research model also for studies on fish nutrition. The zebrafish gastrointestinal anatomy is similar to mammalian small intestine. In zebrafish wild habitats, its diet comprises zooplankton, insect larvae and algae. To maintain zooplankton and insect larvae in the laboratory is not easy, moreover, energy is lost when dried. For these reasons, over the last few decades there has been an increasing interest in the commercial production of food-grade macro- and micro-algae to support growth and increase performance of omnivorous fish (Stanley and Jones, 1976; Wallace et al., 2005; Hardy, 2007; Lawrence, 2007; Spence et al., 2007; Bertuccio, 2013; Marino et al., 2016; Rizzo et al., 2017). Spirulina *(Arthrospira platensis)*, (Spirulinaceae, Cyanobacteria), one of the most commonly used microalgae in aquafeeds, appears to be a good candidate for feeding adult fish. Its use in cichlid farming, in a closed ecological recirculating aquaculture system (CERAS), has been suggested (Lu and Takeuchi, 2004; Guroy et al., 2011, 2012). Furthermore, there is strong scientific interest in Spirulina and its potential therapeutic health benefit as a commercial food supplement and source of potential pharmaceuticals. Spirulina show a unique nutrient blend that no single source can offer, containing several nutrients: amino acids, particularly leucine, valine, isoleucine; essential fatty acids, such as linoleic acid (LA) and γ-linolenic acid (GLA); phycobiliproteins, as phycocyanin and allophycocyanin; β-carotene; E and B-complex vitamins; minerals; and a number of unexplored bioactive compounds. Spirulina lacks cellulose cell walls and therefore is easy to digest (Nakagawa and Montgomery, 2007). The effects of this alga on fish have been investigated in several fish species, including tilapia, *Oreochromis niloticus* (Mustafa et al., 1997; Nandeesha et al., 1998; Takeuchi et al., 2002) white sturgeon, *Acipenser transmontanus* (Palmegiano et al., 2005) and rainbow trout, *Oncorhynchus mykiss* (Matty and Smith, 1978).

This study aims to evaluate the short-term effect of food deprivation and refeeding with a Spirulina diet, on gastrointestinal morphology, as well as on the immunohistochemical expression of PepT1 and hunger hormone GHR, in adult zebrafish (*Danio rerio*) to highlight a potential mechanism driving compensatory growth.

## Materials and Methods

A total of 84 adult (10–12 g) zebrafish (*Danio rerio*), obtained from the Centre for Experimental Fish Pathology of Sicily (CISS), Department of Veterinary Sciences (in accordance with EU Dir 63/2010), were used in this study. To acclimatize to experimental conditions, specimens were stocked for 15 days in 0.4 m^3^ aquarium, filled with dechlorinated freshwater at 25–26°C, pH 7.8 and equipped with a circular water system. These water parameters were used in all trials. Photoperiodic regime was 12L:12D. Zebrafish were fed daily, each morning, with a commercial flake diet (SERA Vipagran, centesimal composition: crude protein 40.4%, crude fat 8.7%, crude fiber 3.4%, crude ash 4.0%, humidity 6.1%; additives per kg: Vit. A 37000 IU, Vit. B1 35 mg, Vit. B2 90 mg, Vit. C 550 mg, Vit. D3 1800 IU, Vit. E 120 mg).

This study used live fish for experimental purposes and was performed under authorization from the Italian Ministry of Health (Prot. CISS 51/2013, DGSAF0022072) according to EU 63/2010 Directive. The *in vivo* study was carried out at CISS, Establishment for Users recognized by the Italian Ministry of Health for experimental activity on aquatic organisms (according to IT D.L. 26/2014), at the Department of Veterinary Sciences. Fish were only fed with commercial pellet supplemented with algae. Water parameters and health status were checked daily. No deaths or pain were caused to fish. At the end of the experimental trial the fish were euthanatized using anesthetics tricaine methanesulfonate (MS-222).

For the experimental trials, fish were divided into 7 groups, each of 12 individuals (**Table 1**). The controls (CF) were fed with a commercial flake diet. Two groups were sacrificed after 2 days (F2) or 5 days (F5) of food deprivation; the remaining groups were refed following the same period of food deprivation as follows: two groups were refed *ad libitum*, twice daily, respectively, for 2 days (F2R2 and F5R2) and further two groups for 5 days (F2R5 and F5R5) with commercial Sera Spirulina tabs, in which Spirulina is the major constituent (centesimal composition of Sera micron manufacture declaration: protein 50.2%, fat 8.1%, moisture 5%, fiber 9.2%, ash 11.9%, and particle size 62 ± 20 μm).

**Table 1:** Experimental Protocol: normal diet, fasting, and refeeding.

Groups	Feeding with Sera Vipagran	Days fasting	Days refeeding with Sera *Spirulina* tabs
CF	Continuous feeding		
F2		2	
F2R2		2	2
F2R5		2	5
F5		5	
F5R2		5	2
F5R5		5	5


**CF: continuous feeding; F2: 2 days fasting; F2R2: 2 days fasting followed by 2 days refeeding; F2R5: 2 days fasting followed by 5 days refeeding; F5: 5 days fasting;F5R2: 5 days fasting followed by 2 days refeeding; F5R5: 5 days fasting followed by 5 days refeeding.**

The experimental protocol was in accordance with the guide for the care and use of laboratory animals and with the Helsinki declaration.

Digestive tracts were dissected under a binocular microscope for future evaluations. The gut was exported from each animal and was fixed in 10% paraformaldehyde in phosphate buffered saline (PBS) 0.1 mol/l (pH 7.4) for 24 h, dehydrated in graded ethanol, cleared in xylene, embedded in Paraplast (McCormick Scientific, St. Louis, MO, United States) and cut into 5 μm triple serial sections. The sections were stained with haematoxylin and eosin (H&E), alcian blue-periodic acid Schiff (AB-PAS; pH 2.5) and May-Grünwald-Giemsa (MGG). Sections were examined under a Zeiss Axioskop 2 plus microscope equipped with a Sony Digital Camera DSC-85.

To better describe cell morphology both immunoperoxidase and immunofluorescence methods were used.

Immunohistochemical investigations were carried out using indirect method of peroxidase with a primary antibody specific for peptide transporter 1 (anti-PepT1 H-235, Santa Cruz Biotechnology, Inc.) and ghrelin (Anti-Ghrelin IN, Z-Fish^TM^, AnaSpec). Serial sections were incubated with 0.3% H_2_O_2_ in PBS pH = 7.4 for 30 min to eliminate endogenous peroxidase activity. They were then rinsed and normal goat antiserum (1:20; Sigma, St. Louis, MO, United States) was added for 60 min. Sections were rinsed and incubated with a primary antibody anti-PepT1 (1:100) and anti-Ghrelin (1:10.000), in a moist chamber at 4°C for 12 h. Following this, the sections were rinsed and incubated for 2 h at room temperature in goat and anti-rabbit immunoglobulins (IgG) peroxidase (HRP) conjugated (1:50; Sigma, St. Louis, MO, United States). The sections were incubated for 1–5 min at room temperature in a 0.02% diaminobenzidine solution (DAB) and 0.015% hydrogen peroxide to visualized peroxidase activity. After rinsing in PBS, sections were dehydrated, mounted, and examined under an Axiophot Zeiss microscope (Carl Zeiss MicroImaging GmbH, Germany) equipped with a Sony Digital Camera DSC-85.

For the immunofluorescence method, serial sections were deparaffinised and rehydrated, rinsed several times in PBS and blocked in 10% normal goat serum for 1 h. Primary antibody anti-PepT1 and anti-Ghrelin were diluted in a permeabilizing solution (PBS, 0.2% Triton X-100, and 0.1% sodium azide) 1:100 and 1:10,000, respectively, placed on slides and incubated overnight in a moist chamber for 12 h at 4°C. Sections were then treated with fluorescent labeled secondary antibody diluted in PBS goat-anti-rabbit conjugated with the Rhodamine Red, Alexa 568 Fluor Dye (Molecular Probes, Invitrogen, Eugene, OR, United States; 1:100) for PepT1 and goat anti-rabbit conjugated with Fluorescent Isothiocyanate (FITC) (Cedarlane Laboratories, Burlington, ON, Canada**;** 1:50) for ghrelin, and left at room temperature for 2 h in the dark. After rinsing, the sections were mounted with Vectashield (Vector Labs, Burlingame, CA, United States) to prevent photo bleaching, and coverslipped. Control experiments were performed excluding primary antibody.

## Results

Fish fed with Sera Spirulina tabs displayed a high survival rate (98%). This diet provided all the necessary components for promoting zebrafish wellness: color, size, quality, as well as attractiveness and availability.

**FIGURE 1:**
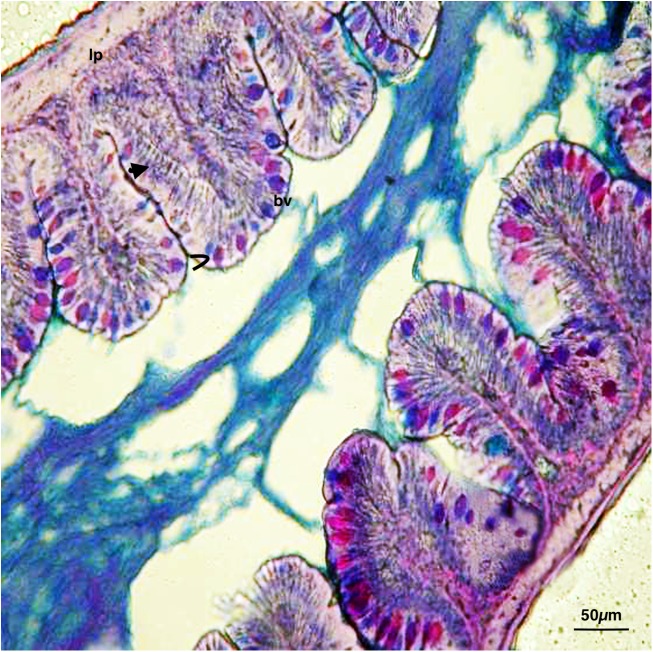
Digestive tract of zebrafish control: Continuous feeding, AB-PAS. 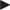, enterocytes; >, goblet cells; bv, blood vessel; lp, *lamina propria*. Scale bars: 50 μm.

The zebrafish has an anterior intestine, called the intestinal bulb, the lumen of which is larger than the lumen of the posterior section, which, similar to a stomach, may function as a reservoir. Intestinal crypts lack a gut epithelial layer; however, folds are present and decreased in size from the anterior to the posterior portion. Absorptive enterocytes (differentiated epithelial cells) (anterior and mid-intestine), mucin-producing goblet cells (entire intestine), and enteroendocrine cells (anterior intestine) were found. Cross-sections (6 μm) of the intestinal segments of control samples, observed with Galgano triple-staining, AB-PAS (**Figure 1**), revealed a simple architecture of the digestive tract. The smooth muscle layer and blood capillaries were directly linked to the mucosa, as the submucosa appeared thinned. Zebrafish enteric nerve cell bodies were present between the smooth muscle layers.

After the 2- and 5-day fasting periods, folds of intestinal mucosa became smaller or fewer and in some sections, linked by anastomosis, obliterated the intestinal lumen. Mucous-secreting goblet cells were decreased in size, number, and intensity. Observations on cross sections of the intestine of specimens refeeding after 2 and 5 days, allowed to reveal the gradual recovery of the fold morphostructure and of the intestinal lumen, respectively. Marked alcianophilia and PAS positivity in the cells of the intestinal mucosa of the samples after 2 days and even more after 5 days of fasting were revealed.

**FIGURE 2:**
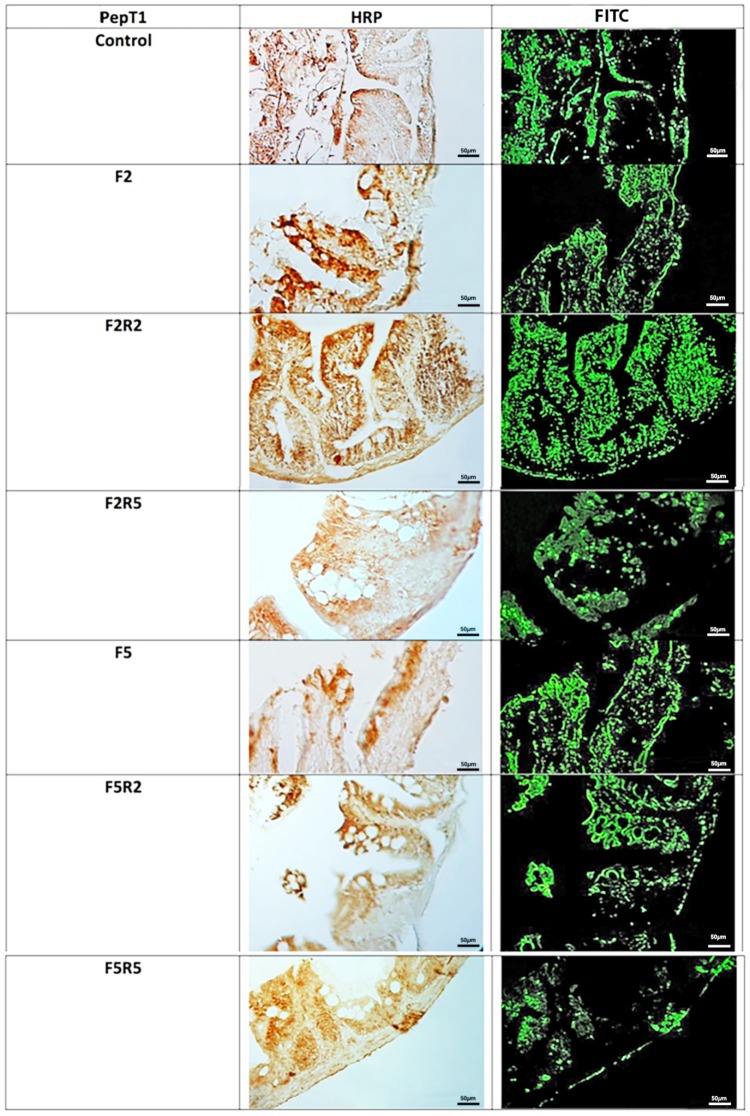
Immunohistochemical detection in HRP and Rhodamine of PepT1 in the digestive tract of zebrafish. Control: Continuous feeding; F2: 2 fasting days; F2R2: 2 fasting days followed by 2 refeeding days; F2R5: 2 fasting days followed by 5 refeeding days; F5: 5 fasting days; F5R2: 5 fasting days followed by 2 refeeding days; F5R5: 5 fasting days followed by 5 refeeding days. Scale bars: 10 μm (F5 and F5R5); 20 μm (F2, F2R2, and F5R2); 50 μm (Control and F2R5).

Immunohistochemical investigation in peroxidase (HRP) and rhodamine-fluorescence, performed with the use of antibody for PepT1 (**Figure 2**), revealed weak immunopositivity in enteroendocrine cells of control samples. After 2 days fasting, immunopositivity decreased compared to controls, increasing after 2 days and even more after 5 days of refeeding with Spirulina. Similar results were observed after fasting for 5 days, and a marked immunopositivity was detected after 5 days of refeeding.

**FIGURE 3:**
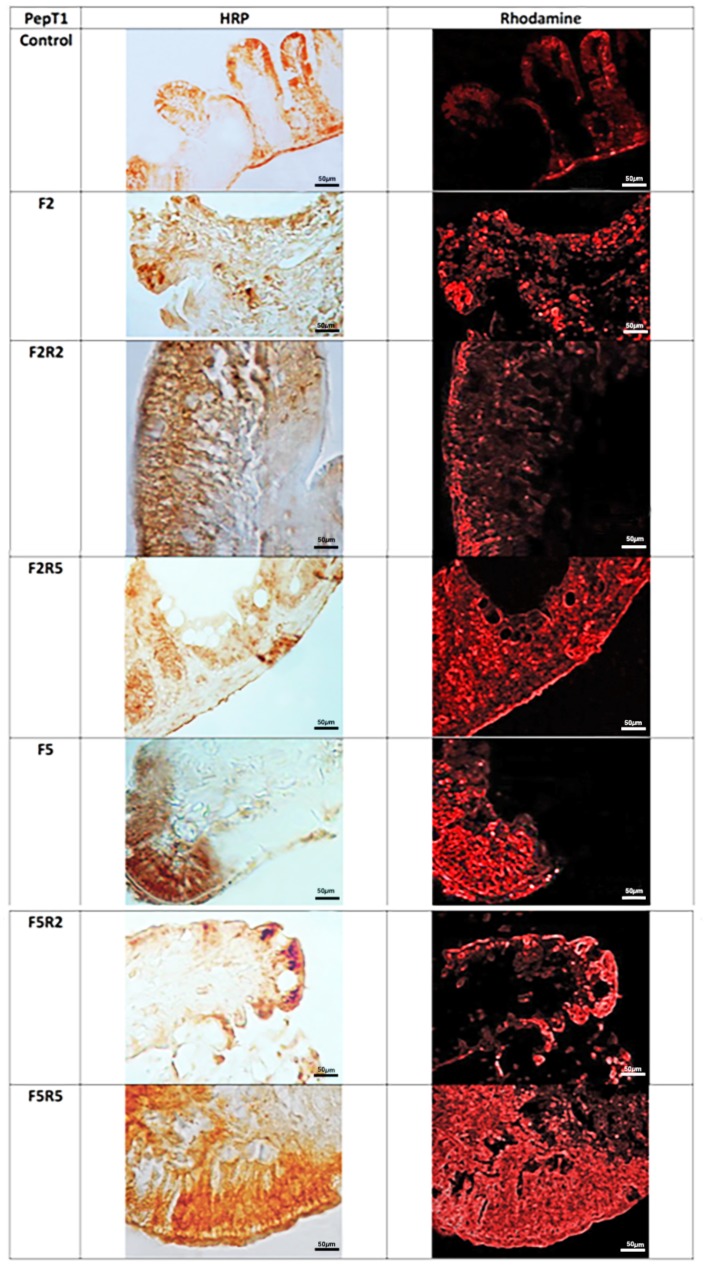
Immunohistochemical detection in HRP and FITC of Ghrelin in the digestive tract of zebrafish. Control: Continuous feeding; F2: 2 fasting days; F2R2: 2 fasting days followed by 2 refeeding days; F2R5: 2 fasting days followed by 5 refeeding days; F5: 5 fasting days; F5R2: 5 fasting days followed by 2 refeeding days; F5R5: 5 fasting days followed by 5 refeeding days. Scale bars: 50 μm.

Immunohistochemical investigation in HRP and Fluorescein Isothiocyanate (FITC) performed with the use of antibody for GHR (**Figure 3**) revealed weak immunopositivity in enteroendocrine cells of control samples. After 2 days of fasting, immunopositivity increased. Refeeding for two and 5 days caused a decrease of immunopositivity, which was particularly marked after 5 days.

## Discussion

In this study, the regulation of PepT1 and GHR after vegetable protein sources were used as an alternative diet of zebrafish, was investigated. In fish subjected to a protocol of fasting of 2 and 5 days followed by refeeding *ad libitum* for 2 and 5 days, using a diet of Spirulina tabs, immunohistochemical analysis showed the levels of PepT1 and GHR compared to the control group. PepT1 levels progressively decreased during food deprivation and increased fourfold after refeeding; instead, GHR levels increased after 2 days of fasting, decreased gradually after refeeding by twice as much as fish fed prior to fasting. PepT1 affinity and its stereoselective capacity varies for different types of peptides, suggesting that its activity may be modulated through diet, particularly using different protein sources (Ostaszewska et al., 2010a,b; Bertuccio, 2013). High rates of several terrestrial plant meals have been successfully included in the feed without influencing fish growth and production quality (Carter and Hauler, 2000; Aslaksen et al., 2007; Sánchez-Lozano et al., 2011). The high level of PepT1 conservation through evolution is not only consistent with its important role in growth and metabolism, but also suggests that its biological action may be well conserved. Therefore, numerous aspects of digestion and absorption in fish and mammals are similar, demonstrating high conservation of these mechanisms during evolution, in humans (Ford et al., 2003) and fish species such as Atlantic cod, sea bass (Rønnestad et al., 2007; Terova et al., 2009), weather loach (Gonçalves et al., 2007), zebrafish (Verri et al., 2003), and sea bream (Lauriano et al., 2012). mRNA transcription and activity of Pep T1 have been shown to increase to compensate absorptive capability in patients with short-bowel syndrome chronic ulcerative colitis and with Crohn’s disease (Merlin et al., 2001; Ziegler et al., 2002). A similar strategy may be active in fish that exhibit compensatory growth following a period of food deprivation (Navarro et al., 1997; Belanger et al., 2002). In support of this, it has been shown that in sea bass (*Dicentrarchus labrax*) Pep T1 was involved in compensatory growth during refeeding after a period of fasting (Verri et al., 2011). These mechanisms also influence gastrointestinal hormone levels such as cholecystokinin, gastrin-releasing peptide, GHR (Darcel et al., 2005; Liou et al., 2011). GHR is involved in the regulation of appetite and feeding in vertebrates. There is evidence that GHR functions in energy homeostasis control and food intake increase (Volkoff and Peter, 2006). GHR presence increases in the hypothalamus and gut of goldfish and in the stomach of sea bass with food deprivation (Terova et al., 2008). In this study, GHR was mainly found in the intestinal tract of zebrafish after 2, and much more after 5, days of fasting. Similar to studies of other fish species without a stomach, such as goldfish and common carp, GHR was predominantly found in the gut (Unniappan et al., 2002). Elevated GHR levels in the intestine of blunt snout bream may be related to its biological regulation of appetite. For fish species with stomachs, such as rainbow trout (Kaiya et al., 2003), sea bass (Terova et al., 2008), and Atlantic cod (Xu and Volkoff, 2009), GHR was firstly detected in the stomach. In conclusion, fasting and refeeding experiments confirmed that GHR has the opposite function to PepT1 and CCK, involved in the regulation of feeding in zebrafish. These findings give a further contribution to understanding the role of GHR, in the regulation of zebrafish appetite and could be useful for providing basic information for a responsible aquaculture of teleosts.

The results confirm that the role of PepT1 and GHR is of major nutritional significance for intraluminal products of protein digestion. Although there are many differences between teleosts and mammalian intestines, this paper confirms high conservation of these physiological mechanisms during evolution.

## Author Contributions

PL and CC: protocol design and manuscript writing. CB: experimental challenge and immunohistochemistry. CI: animal care and histology. FM: manuscript editing and animal welfare. MD: data analysis.

## Conflict of Interest Statement

The authors declare that the research was conducted in the absence of any commercial or financial relationships that could be construed as a potential conflict of interest.
